# A clinical case of orbital inflammatory pseudotumor as the primary expression of eosinophilic angiocentric fibrosis

**DOI:** 10.22336/rjo.2021.82

**Published:** 2021

**Authors:** Helen-Melissa Heedari, Andra-Carmina Ciotoracu, Traian Costin Mitulescu, Monica-Gabriela Dimancescu, Simona Enache, Denisa Predețeanu

**Affiliations:** * Department of Rheumatology and Internal Medicine, “Sfânta Maria” Clinical Hospital, Bucharest, Romania; ** Department of Ophthalmology, Emergency University Hospital, Bucharest, Romania; *** Department of Ophthalmology, “Sfânta Maria” Clinical Hospital, Bucharest, Romania; **** Pathological Anatomy Laboratory, “Sfânta Maria” Clinical Hospital, Bucharest, Romania; ***** “Carol Davila” University of Medicine and Pharmacy, Bucharest, Romania

**Keywords:** orbital pseudotumor, eosinophilic angiocentric fibrosis, proptosis

## Abstract

Eosinophilic angiocentric fibrosis (EAF) is an infrequent and slowly progressive disease, represented by fibroinflammatory lesions of unknown origin, which mainly involves the sinonasal structures and upper respiratory tract. Occasionally, it can affect the orbit and ocular adnexa causing symptoms such as proptosis, globe displacement and periorbital edema. In very rare cases, ocular manifestation as an orbital inflammatory pseudotumor can be the primary localization of the disease. Current literature proposes a relation between EAF and immunoglobulin G4-related disease spectrum.

We describe the case of a 69-year-old man presented with antecedents of left periorbital edema, epiphora and retroocular pain. Examination showed a nonaxial proptosis, severe limitation in left eye adduction and lateral globe displacement. Orbital imaging revealed a left medial orbital mass with involvement of the inferior rectus and the medial rectus muscles. An orbital biopsy of the mass illustrated an inflammatory infiltrate with a notable eosinophilic component, “onion-skin appearance” of vessels and surrounding concentric fibrosis, highly suggestive of EAF. Further investigations showed a high expression of IgG4 and excluded other possible diseases. There was a favorable evolution of the orbital inflammatory pseudotumor following a 4-month treatment course with oral glucocorticoids.

**Abbreviations:** EAF = Eosinophilic angiocentric fibrosis, CT = Computed tomography, MRI = Magnetic resonance imaging, GPA = Granulomatosis with polyangiitis, EGP = eosinophilic granulomatosis with polyangiitis, MPA = microscopic polyangiitis, ANCA = Anti-neutrophil cytoplasmic antibodies, Ig = Immunoglobulin

## Introduction

Eosinophilic angiocentric fibrosis (EAF) is an unusual tumefactive lesion of the orbit, sinonasal structures, and upper respiratory tract. The inflammatory infiltrate within this lesion is an admixture of predominantly eosinophils along with lymphocytes and plasma cells. The characteristic histologic feature of EAF is the appearance of small-caliber arteries surrounded by concentric layers of fibrosis, also reported as “onion-skin” appearance. Little has been discovered about the etiology of EAF since its first description in 1985 [**1**].

It predominantly affects the sinonasal tract, the larynx and the gums and infrequently the orbit and ocular adnexa. In limited cases, ocular manifestation can be the primary localization of the disease [**2**]. This benign disease is represented by a gradually progressive process imitating a tumor, with many recurrences after surgical extirpation and glucocorticoid therapy. The typical histology is made up of fibro-inflammatory lesion with several eosinophils, organized in a perivascular pattern. As the lesion evolves, inflammation becomes not so much intense and the fibrosis appears like “onion-skin” type perivascular fibrosis.

Deshpande et al. found a patient with EAF with a significant increase of serum IgG4. This unintentional finding led to a retrospective analysis of 5 EAF cases and proposed the probability that EAF would be a manifestation of IgG4-related disorder [**3**].

Not too many EAF cases with nasolacrimal duct obstruction and orbital involvement have been described in literature. These cases described patients with periorbital edema, globe displacement, painless proptosis, epiphora, diplopia and limitation in ocular movements, on physical examination [**2**,**4**,**5**].

We describe a case of orbital EAF related with the existence of IgG4-containing plasma cells in the orbital biopsy sample of a man, which sustained this hypothesis. The main motivation of this case report was to raise awareness about this rare entity, to review current literature and to discuss its clinical and histological features that guide the diagnostic process as well as its management options.

## Case report

**Fig. 1:**
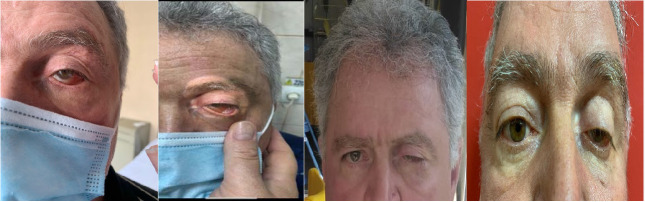
Left periorbital edema and eyelid ptosis - different stages of evolution under treatment

We report the case of a 69-year-old man who presented with a 3-year history of left orbital inflammatory pseudotumor, periorbital edema, epiphora and retroocular pain. He had chronic sinusitis with multiple sinus surgeries over a 4-year period. Due to the orbital involvement of the left eye, the patient was suspected of GPA and was referred to the Rheumatology Department for further investigations and specialized treatment. The clinical examination was normal. On physical ophthalmologic examination, visual acuity was normal; there was a moderate left periorbital edema, but no tenderness or erythema. A left nonaxial proptosis and lateral globe displacement was noted, as well as severe limitation of left eye adduction (**Fig. 1**).

The full blood count was normal, with no peripheral eosinophilia. A full autoimmune screen (antinuclear antibody, p-ANCA, c-ANCA, anti-Ro/ SSA, anti-La/ SSB antibodies, rheumatoid factor) was negative and did not provide any evidence of an autoimmune disorder. All other blood test results, including serum chemistry profile, serum IgG, IgA, IgM, C-reactive protein and erythrocyte sedimentation rate were normal; serum IgG4 level was also in normal range.

**Fig. 2:**
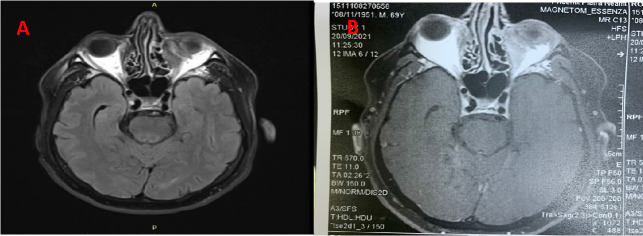
MRI of the orbital inflammatory pseudotumor - before and after treatment (A. 41/ 20/ 15 mm; B- 31/ 10/ 12 mm)

**Fig. 3:**
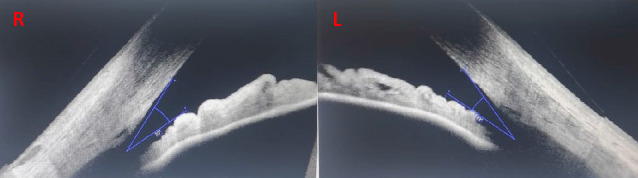
Gonio-OCT - Iridocorneal angle (Right eye - 23 grades, Left eye - 19 grades)

**Fig. 4:**
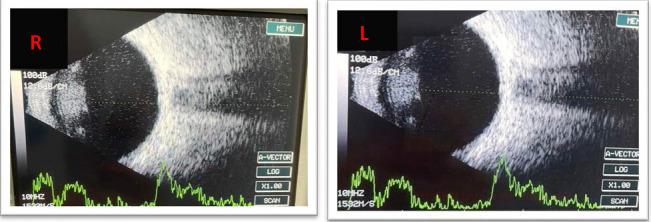
Ocular echography - minimal vitreous echoes with degenerative character

Magnetic resonance imaging (MRI) showed a moderately enhancing left medial orbital mass eroding the medial orbital wall and involving the inferior rectus and the medial rectus muscles (**Fig. 2**-**4**).

**Fig. 5:**
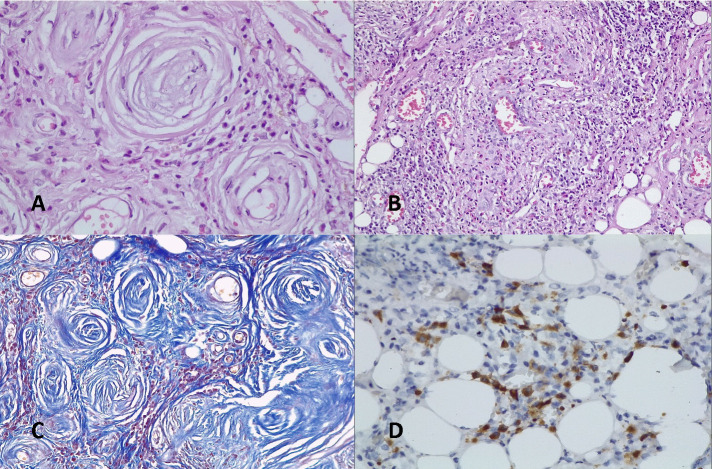
**A.** HE 40X “onion skin” type fibrosis around blood vessels; **B.** HE 20X marked inflammatory cells with many eosinophils; **C.** Trichrome Masson 20X fibrosis “onion skin” type surrounding small blood vessels; **D.** IgG4 20X positive in ~40-45% of inflammatory cells

The histopathological appearance of the left orbital biopsy revealed heavy concentric fibrosis, polymorphous inflammatory infiltrate including plasma cells, histiocytes, lymphocytes and frequent eosinophils, featuring the “onion-skin” like perivascular whirling pattern. There was no distinct granulomatous inflammation or necrosis. Immunohistochemistry staining showed that IgG4 staining was mainly positive and IgG4 plasma cells were ~ 40% of all the inflammatory cells (**Fig. 5**).

The histologic findings from the left orbital biopsy were the classic features of EAF. The absence of the classic necrotizing or granulomatous areas typically encountered in GPA, the presence of the characteristic lamellar “onion-skin” appearance of angiocentric fibrosis along with the immunohistochemistry staining that showed a high percentage of IgG4 positive plasma cells established the diagnosis of EAF.

 The therapy was immediately initiated and it consisted of methylprednisolone (starting dose 48 mg/ day, tapering 8 mg every two weeks) for over 4 months. Throughout this time, our patient had a favorable evolution consisting of decreased periorbital edema, without improvement of the ocular motility.

## Discussion

We presented a patient with EAF, which primarily affected the orbit, resulting in globe displacement, proptosis, and limitation in ocular movements. 

The term “EAF of the upper respiratory tract” was used by Roberts and McCann to describe a condition characterized by inflammatory cell infiltration, mainly with eosinophils and gradual perivascular fibrosis [**1**]. In 2011, EAF was described as being component of the immunoglobulin G4-related condition spectrum [**3**]. IgG4-related diseases are a group of inflammatory conditions that affect many organs of the body, including the pancreas, lungs, kidneys, and salivary glands. Serum IgG4 is only elevated in 50% of patients. The diagnosis is generally based on histology. A high expression of IgG4-related plasma cells and an IgG4:IgG ratio > 0.4 in the biopsy sample support the diagnosis [**6**].

EAF symptoms are nonspecific. The most common symptoms are related to the sinonasal region consisting of progressive nasal obstruction, sinusitis, swelling, nasal tenderness, facial pain, and shortness of breath [**4**,**7**-**9**].

Imaging, both CT and MRI, is nonspecific and usually describes a well-circumscribed submucosal tissue density mass with sinus opacification [**6**]. EAF is a progressive disorder, but there is some proof that the lesion stabilizes over time, and no EAF-associated mortalities have been documented. 

Histopathological examination shows both early and late lesions. The early lesion is a vasculitis with inflammatory infiltrate containing lymphocytes, plasma cells, scattered neutrophils and with numerous eosinophils. The late lesions consist of heavy fibrosis with angiocentric pattern and combined inflammatory cells. The fibrous component has a concentric stratified “onion-skin” type perivascular layout, which is typical for EAF and is useful for establishing the histologic diagnosis of EAF [**1**,**2**,**7**,**10**,**11**].

The differential diagnosis of EAF is extensive and includes the ANCA-associated vasculitis spectrum with its three major entities (GPA, EGP and MPA), two neoplastic conditions represented by NK/ T cell lymphoma and lymphomatoid granulomatosis, infectious granulomatous diseases, sarcoidosis, Sjogren’s syndrome, etc. [**8**,**9**,**11**].

The limited form of GPA is an important entity, with heterogenous clinical and pathological manifestations and should be considered in the differential diagnosis of EAF [**12**,**13**]. In both entities, ANCA tests can be negative, and considerable numbers of eosinophils may be seen in the histopathology report. Our patient did not have the classic necrotizing granulomatous vasculitis of typical GPA, but did have striking lamellar “onion-skin” angiocentric fibrosis with a low-grade vasculitis. The advanced angiocentric fibrosis suggests a well-established chronic lesion. Eosinophils and fibrosis can be seen in early cases of orbital GPA, but rarely in chronic lesions. Moreover, the lack of typical histologic lesions of GPA makes this diagnosis unlikely [**14**].

The initial step in managing patients who have an orbital inflammatory pseudotumor is to exclude alternate inflammatory and neoplastic diseases based on the clinical and histological findings [**9**]. All patients should undergo a thorough blood examination (full blood count, erythrocyte sedimentation rate and complete serological markers for rheumatic diseases). In addition, the patients should be referred to a rheumatologist to exclude any rheumatologic diseases and to an ear, nose, and throat specialist to exclude any nasal or sinus involvement. Once the diagnosis of EAF is established, the possible treatments are medical or surgical. Surgical resection seems to be the treatment of choice in most previously reported cases. However, recurrences are very frequent and repeated excisions are usually required to control the sometimes-slow evolution of the disease [**2**,**9**,**15**-**17**].

Glucocorticoids had beneficial effect in our case over a 4-month treatment course.

## Conclusion

EAF is a benign fibrosing lesion affecting the sinonasal tract and infrequently extends to the orbit. The clinical manifestations of EAF with orbital involvement often mimic other more common ophthalmological diseases. The physical, laboratory and radiological findings of these patients are commonly nonspecific, thus requiring more extensive investigations. In this circumstance, biopsies of the involved lesions can be revelatory in the process of finding the diagnosis. Also, the response to glucocorticoids depends on the histological stage (inflammatory or fibrotic) of the lesion. In our case, we expected a slowly favorable progression of the disease due to the predominantly fibrotic histological phase of the orbital inflammatory pseudotumor. Even if EAF is very uncommon in the upper airway tract, nasal cavity, and orbit it should be taken into consideration in the differential diagnosis of lesions of these regions.

**Conflict of Interest statement**

Authors state no conflict of interest.

**Informed Consent and Human and Animal Rights statement**

Informed consent has been obtained from all individuals included in this study.

**Authorization for the use of human subjects**

Ethical approval: The research related to human use complies with all the relevant national regulations, institutional policies, is in accordance with the tenets of the Helsinki Declaration, and has been approved by the review board of “Sfânta Maria” Clinical Hospital, Bucharest, Romania.

**Acknowledgements**

None.

**Sources of Funding**

None.

**Disclosures**

None.
